# Role of Supplemental Teriparatide Therapy to Augment Functional and Radiological Outcomes in Osteoporotic Intertrochanteric Hip Fractures in the Elderly Population

**DOI:** 10.7759/cureus.26190

**Published:** 2022-06-22

**Authors:** Sanket Mishra, Deepankar Satapathy, Sidhartha Samal, Nego Zion, Udeepto Lodh

**Affiliations:** 1 Department of Orthopedic Surgery, Institute of Medical Sciences & SUM Hospital, Bhubaneswar, IND

**Keywords:** intertrochanteric fracture, bone density, osteoporotic fracture, fracture union, teriparatide

## Abstract

With improved life expectancy and ever-increasing geriatric population with concomitant osteoporosis, there is increase in osteoporotic intertrochanteric hip fractures. Even the best surgical advances fail to provide satisfactory and early results. As a result, researchers' focus has lately shifted to developing a more integrated approach that combines the pharmacotherapeutic capabilities of teriparatide, a recombinant version of human parathyroid hormone (1-34), a new anabolic drug that enhances bone mass and strength by promoting osteoblastic activity and hastens fracture union in both human and animals. We attempted to evaluate the therapeutic efficiency of teriparatide therapy on outcomes of surgically managed Intertrochanteric hip fractures in osteoporotic patients. A total of 31 patients with established osteoporosis and Intertrochanteric fractures were selected and divided into two groups, managed surgically with proximal femur nailing, and then prospectively compared with one group receiving teriparatide therapy in addition to standard treatment after taking necessary consent and allocation into two groups based on the preference of patients to take additional teriparatide or not after understanding the benefits and risks involved. We aimed to assess the functional and radiological effects of teriparatide on bone mineral density, the time taken for fracture union, and other fracture-related postoperative complications such as weight bearing and residual bone pain. All patients were followed up at 6, 12, and 24 weeks. Time to fracture union was significantly shortened, with considerable improvement in bone density and functional outcome in the teriparatide group. Varus collapse, the rate of migration of the helical blade, and shortening of the femoral neck were also significantly less in the study group. From the assembled data, we can safely assume that with early union rates with better functional improvement with additional advantage of increased bone mass, we favor supplemental teriparatide therapy in the management of osteoporotic patients with femoral intertrochanteric fractures to augment healing. Further studies with a larger sample size are required to support our observation.

## Introduction

Improving living conditions and healthcare facilities worldwide has improved life expectancy, leading to a steep rise in the geriatric population with subsequent rise in geriatric trauma [1]. Among traumatic geriatric injuries, intertrochanteric femur fractures are one of the commonest injury around the hip. Around 5.6% of males and 20% for females population at 50 years of age are at a risk of sustaining hip fractures [2]. Minor and trivial trauma has been the major etiology for such fractures, with 53% of such fracture in those <50 years and 80% in those >80 years attributed to such trivial trauma [3]. Osteoporosis is a metabolic condition fairly well established at such ages, frequently predisposing to such fractures and often complicating post-fracture recovery, leading to high morbidity, notable functional loss, and poor quality of life in the elderly people, and sometimes resulting in higher mortality rate in these patients [4-5].

Surgical fixation is considered standard treatment for such pertrochanteric fractures. But pain relief, early weight bearing, and mobilization for early return to day-to-day activities are critical factors in these groups of patients to improve their physical and psychological conditions [6]. These factors create challenges for modern-day orthopedic surgeons. Even after adequate fixation, satisfactory reduction, and implant positioning, stability largely depends on bone quality and fracture comminution that subsequently leads to failure in the geriatric population because of reduced bone stock and regenerative capacity of the bone [7]. New implants have evolved over time, but these new designs are also aimed at enhanced purchase and holding fracture reduction for appropriate time till the bones unite, but such advanced designs also have no control on bone quality and are subsequently at the mercy of biological bony union, thus not free from failure [8].

In the recovery phase, after fixation of osteoporotic fractures, most patients are left with chronic pain, reduced mobility, and impairment of daily activity of living with a large population unable to achieve functional level at or equal to pre-fracture state even after one year of follow-up [9].

Osteoporosis is a generalized systemic condition, characterized by a progressive reduction in bone mass and the breakdown of bone matrix and micro architecture, leading to an increased vulnerability for fractures [10]. If a multimodal strategy with a combination of surgery and pharmacotherapy be used, there is a scope to improve fracture union that would potentially accelerate recovery, help in early ambulation, help in quick return to activities, and also decrease the chances of revision surgery [11].

Drugs used for the management of osteoporosis are broadly categorized into two main types: antiresorptive and anabolic drugs. The positive effect of these drugs on bone turnover and increase in bone mass in diagnosed cases of osteoporosis has been demonstrated clinically by a reduction in fracture incidence in treated patients and also by bone mineral density (BMD). Antiresorptive agents include a wide range of drugs such as bisphosphonates, calcitonin, raloxifene, denosumab, and HRT (hormonal replacement therapy), which act by inhibiting bone resorption [12]. Anabolic drugs, on the other hand, stimulate progressive bone formation by causing an increase in bone remodeling through their action on osteoblast cells, resulting in increasing bone mineral content, increased cortical thickness, and enhanced fracture healing [13]. This group includes teriparatide, which is a recombinant form of biologically active fragment of human parathormone (human parathyroid hormone [PTH] 1-34). FDA approved it for the management of osteoporosis in the year 2002 [14]. It is conceptualized to work on theory of anabolic window, which simply means that teriparatide stimulates bone formation process earlier than bone resorption [15]. Various animal trials with teriparatide have shown its positive ability to improve fracture union and to promote callus volume, bone mineralization, and strength [16]. This has encouraged trials in humans. In pre-clinical and controlled clinical trials, teriparatide has been shown to enhance fracture healing [17]. Meta-analysis reports and literature suggest that daily subcutaneous teriparatide enhances bone healing and improves functional recovery after fragility fractures at regions including the spine, hip, and distal radius [18]. But most of these trials are limited to the western population with limited data and limited duration of therapy (average 12 weeks), where the demographics, nutrition, body mass, and bone density are fairly different in contrast to the Asian and Indian populations.

In our study, we aim to study and evaluate the effect of daily subcutaneous teriparatide therapy for 24 weeks on the rate and time of fracture healing in the elderly with osteoporotic intertrochanteric fractures in the Indian population. In addition, functional outcome, improvement in BMD, implant stability and migration, and complications were also analyzed.

## Materials and methods

After necessary institutional ethical clearance (DRI/IMS.SH/SOA/2021/061), a prospective randomized controlled study was conducted at IMS & SUM Hospital, Bhubaneswar from January 2021 till December 2021. All the patients included in the study were above the age of 50 presenting with isolated unilateral Intertrochanteric femur fracture with established osteoporosis on DEXA scan. A score of -2.5 or lower was considered as established osteoporosis, and such patients were included in the study. The surgical plan and choice of implant was consistent for all cases and consisted of closed intramedullary nailing using proximal femoral nailing (intramedullary nail with a single helical blade made of titanium) from a single manufacturer, and the surgery was performed by the same group of surgeons.

All routine blood investigations were done to rule out other metabolic conditions that could significantly affect the outcomes, and any such patients were excluded from the study. Exclusion criteria included (1) patients not giving consent (2) patients allergic to teriparatide or any previous drug allergy history, (3) patients with a history of previous use of teriparatide or antiresorptive drugs, (4) patients with severe comorbidities with poor life expectancy, (5) patients with other associated metabolic disorders, (6) patients with DEXA score of > -2.5 at presentation, and (7) patients with multiple fractures.

Following strict ethical guidelines and after obtaining written consent of all patients, detailed history was noted, and general physical and systemic examination were performed, following which the patients were subjected to relevant investigations. Patients with low BMI (<18.5) were considered underweight and not selected for study as the nutrition quality and habits of these patients were questionable and could have affected outcomes. Patients selected for the study were informed about the surgical procedure and postoperative medications and rehabilitation programs, and their informed consent was sought regarding their willingness to be administered the drug teriparatide in addition to standard medications after explaining its possible role and side effects. Those who refused to take additional teriparatide were kept in group A, while patients willing for the additional teriparatide were kept in group B. Both groups were kept completely separate from each other with no access to each other’s treatment, medical reports, or demographic details. A team of nurses and paramedics who were unaware of the study supervised the patients’ progress daily in addition to the surgical team. Group A received only calcium and vitamin D3 supplements in addition to standard postoperative medication, which included antibiotics, analgesics, and proton pump inhibitors, while group B received injection teriparatide 20 micrograms subcutaneous over the anterolateral aspect of the thigh from day 5 onward, in addition to calcium, vitamin D3, and standard postoperative medication. Same brands of calcium, vitamin D3, and other medications were used in both groups. In group B, the same brand of injection teriparatide was used for all patients. Both groups were closely observed for any drug-related side effects especially group B, including injection site reactions, muscle cramps, and behavioral changes.

Additional blood tests included serum PTH, serum Ca+2, serum phosphate, and serum vitamin D levels. PTH levels were measured using the serum electrochemiluminiscence immunoassay (ECLIA) method with a biological reference range of 15-65 pg/ml. The collected data were noted in a specially designed form. AO/OTA (AO Foundation and Orthopaedic Trauma Association) classification of fracture was used to classify the fractures. Type 31A1 (stable) and types 31A2 and 31A3 (unstable) both were included in study. Eligible patients after preanesthetic evaluation were planned for surgery and were treated with proximal femoral nailing. All patients underwent radiographic examinations including an anteroposterior (AP) view of the pelvis with both hips, AP view, and lateral views of the affected hip with zero magnification preoperatively, postoperatively at day 2, at 6 weeks, at 12 weeks, and at 24 weeks.

Intraoperatively, quality of reduction was checked fluoroscopically and considered acceptable if the fracture gap was <5 mm and varus-valgus angulation was <10° and/or anteversion-retroversion was <10°. The helical blade was considered to be ideally placed if it was central-central quadrant in AP and lateral views. Position in inferior-central quadrant or central-posterior quadrant was also accepted.

**Figure 1:**
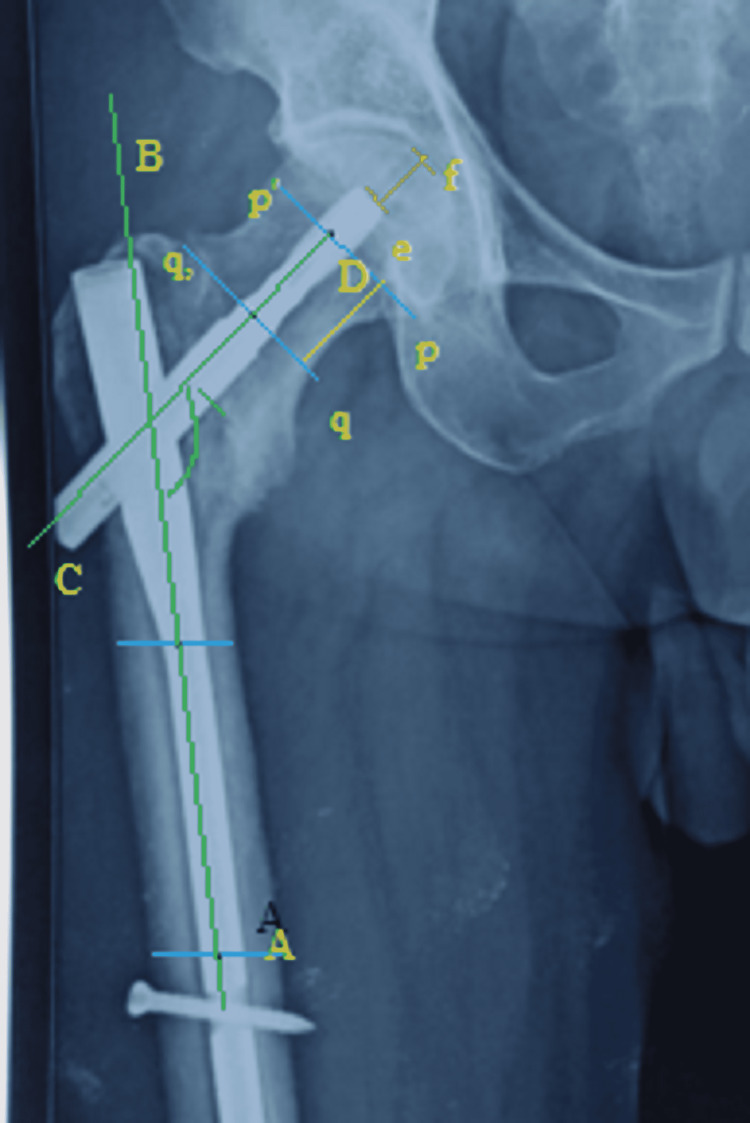
Hip X-ray on zero magnification. Line AB was drawn by joining the midpoint of two imaginary parallel lines drawn on the shaft. Similarly, line CD was drawn by joining midpoints of two lines pp’ (drawn at and parallel to subcapital region) and line qq’ (drawn at basicervical region and parallel to pp’). Angle formed by intersection of line AB and CD gives the neck shaft angle. The distance between p and q gives length of the femoral neck. Distance ef in AP view plus lateral view gives the tip-apex distance.

Postoperative zero magnification AP\lateral X-rays were obtained on day 2, and X-rays of the neck shaft angle, tip apex distance, and length of femoral necks were obtained by simple novel method, as shown in Figure 1. Migration of tip of the blade was assessed according to the progressive changes in the tip apex distance on serial X-rays. Varus collapse was assessed by the alterations in neck and shaft angle, in AP view radiographs. Physiotherapy was advised from the second postoperative day with quadriceps and knee bending exercises as per pain tolerance. Toe touch weight bearing with a walker was advised from 20th postoperative day onwards, and full weight bearing after six weeks assisted by walker and then after 10 weeks nonassisted weight bearing was allowed in most cases.

Clinical follow-up patients were evaluated with blood investigation, DEXA scan, X-ray, and clinical examination at 6 weeks, 12 weeks, and 24 weeks. Radiographic union of the fracture was defined as visible bridging callus, recanalization of the trabeculae, cortical continuity and fracture line disappearance, visible on both radiographic views, and was evaluated using the Radiographic Union Score of Hip (RUSH) score in a separate printed scoring form. The RUSH score is a checklist-based novel scoring system for hip fractures, which was developed analogous to the Radiographic Union Score for Tibial fractures (RUST) checklist [19]. Bhandari et al. initially used the RUSH checklist to assess hip fracture healing using a single radiograph where reviewers were unaware of the time from surgery for each radiograph [20]. Further valgus-varus angulation and tip apex distance and length of the neck was noted with the said methods. Functional outcome of the patients was obtained using the Palmer and Parker mobility score and was recorded in a printed mobility questionnaire form [21]. The data collected were entered into a master chart and subjected to statistical analysis by the biostatistician of our institution.

A total of 32 patients were selected in the study, with 16 in each group A (nonteriparatide) and group B (teriparatide). One patient of group A died in course of treatment and thus was finally not accounted in the study statistics. The observations were recorded and analyzed in Microsoft excel software (2019 version) using unpaired t-test. A p-value of <0.05 was considered significant for the study. Variation in data was expressed in terms of standard deviation (SD).

## Results

The 31 patients randomly allocated into group A and group B were almost similar in terms of age, gender, and body mass index. There was some variation in distribution of individual fracture types in both groups, but it was statistically insignificant. Details are described in Table 1.

**Table 1:** Demographic details

	Group A (mean±SD)	Group B (mean±SD)	p-Value
Age of patient undergoing surgery	72.6±0.25	72.88±1.24	0.460
Gender			
Male	5	7	
Female	10	9	
Body mass index (kg/m^2^)	22.83±1.48	23.06±0.24	0.62
AO classified fracture			
A1 (stable)	7	6	0.45
A2, A3 (unstable)	8	10	0.47

There was significant improvement in fracture union time noted in the study group. Although all the 31 patients achieved union by 24 weeks, and no union was seen at 6 weeks in both groups, the union rate was only 13.33% (two patients) in group A by 12 weeks, which was lower than that in the study group B, where the union rate was 56.25% (nine patients). This shows a statically significant improvement in fracture healing rates with teriperatide treatment (p=0.041) (Table 2). The distance from the tip of the helical blade plate till the medical cortex of the femoral head was measured in both AP and lateral views in millimeters. This tip-apex distance was measured at each follow-up. A reduction in tip-apex distance indicated migration of helical blade plate progressively as the patient started to bear weight. The tip-apex distances of both groups were similar till 12 weeks of follow-up. But by 24 weeks, the tip-apex distance in group A was 22.57 ± 1.33, which was significantly much less than that in group B at 24.26±1.92 (p=0.031), suggesting earlier bony union and consolidation in the teriperatide group, which prevented blade migration, thus substantially reducing chance of screw cutout, hip pain, and implant failure (Table 2). The change in the femoral neck shaft angle was recorded, and an increase in varus angulation was noted. The varus changes were comparable in both groups upto 12 weeks. But, at 24 weeks, the varus angulations in group B was 4.94±1.9 degrees, which was significantly more than that in group B, with a varus angle of 2.86±1.36 (p=0.03). More varus collapse could have been a result of delayed bone union as the patient started weight bearing and thus putting the implant under stress, which resulted in increased stress over the calcar and medial femoral cortex. The length of the femoral neck was also measured each time, and at 24 weeks significant shortening was observed in group A at 7.02±3.43 mm in comparison to 5.13±2.41mm in group B (p=0.02).

**Table 2:** Follow-up outcome data

	Group A (mean±SD)	Group B (mean±SD)	p-Value
Fracture union			
6 weeks	0	0	
12 weeks	2 (13.33%)	9 (56.25%)	0.041
24 week	15 (100%)	16 (100%)	
Tip-apex distance (in mm) (measure of blade migration)			
6 weeks	25.8±3.36	26.27±5.87	0.51
12 weeks	23.96±5.41	25.74±3.35	0.36
24 weeks	22.57±1.33	24.26±1.92	0.031
Change in neck shaft angulation (varus collapse) (in degree)			
6 weeks	0.62±1.03	0.24±0.76	0.15
12 weeks	3.52±1.24	1.91±1.57	0.06
24 weeks	4.94±1.90	2.86±1.36	0.03
Femoral neck length shortening (in mm)			
6 weeks	0.15±0.72	0.09±0.06	0.38
12 weeks	3.72±2.1	3.22±1.91	0.53
24 weeks	7.02±3.43	5.13±2.41	0.02

**Figure 2:**
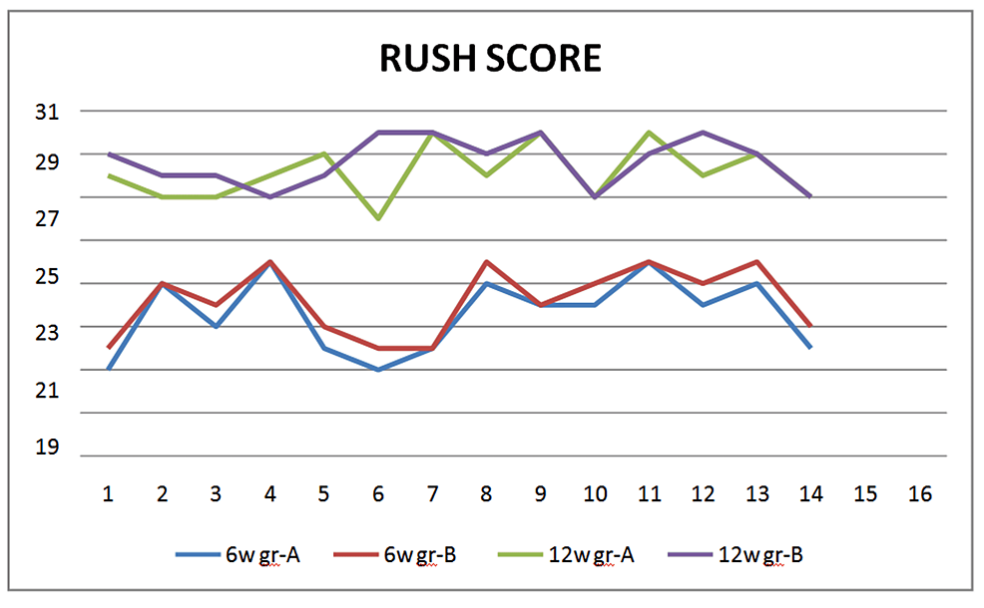
Line chart showing variations in the RUSH score at the 12- and 24-week follow-up RUSH, Radiographic Union Score of Hip

Radiological assessment done using the RUSH score revealed a score of 0 to start with as there was no evidence of union, and it was 30 in both groups by 24 weeks, suggesting complete radiological union in either group. Comparable scores of 21.53±2.83 and 22.2±2.31 were obtained by six weeks in group A and group B, respectively. But by 12 weeks, group B had a score of 28.6±1.25, which was statistically much better than group A with a score of 28.03±1.55 (p=0.019), indicating earlier evidence of radiological union in the teriparatide group (Table 3) (Figure 2).

**Table 3:** RUSH score and Palmer and Parker score RUSH, Radiographic Union Score of Hip

	Group A (mean±SD)	Group B (mean±SD)	p-Value
RUSH score			
6 weeks	21.53±2.83	22.2±2.31	0.0625
12 weeks	28.03±1.55	28.6±1.25	0.019
24 weeks	30	30	-
Palmer and Parker score			
6 weeks	2.46±1.12	2.6±1.25	0.408
12 weeks	6.53±1.12	6.86±1.26	0.0458
24 weeks	8.6±0.17	8.86±0.12	0.0022

**Figure 3:**
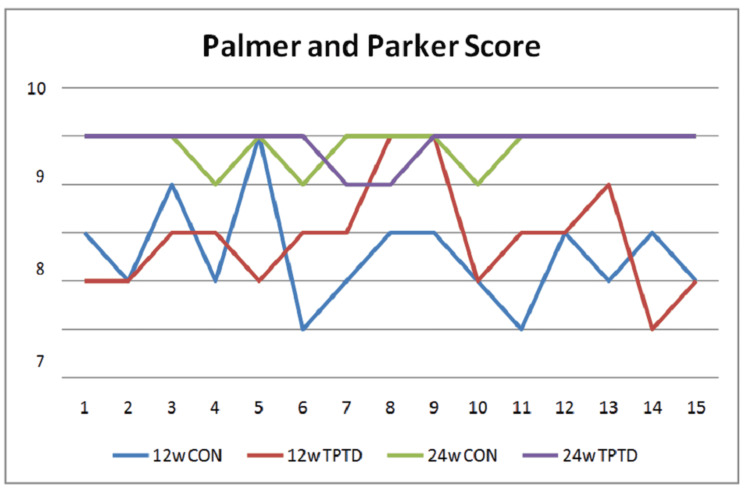
Line chart showing variations in the Palmer and Parker score at the 12- and 24-week follow-up

Functional scoring, which was done using the Palmer and Parker mobility scoring, showed comparable outcomes by six weeks with a score of 2.46±1.12 and 2.6±1.25 in group A and group B, respectively, but these scores improved significantly by 12 weeks to 6.86±1.26 and by 24 weeks to 8.86±0.12 in group B in comparison to group A with scores of 6.53±1.12 and 8.6±0.17, respectively (p=0.045 and 0.002, respectively), suggesting much improved functional outcomes secondary to improved bone density and earlier union (Table 3) (Figure 3).

The bone density was also measured periodically using the DEXA scan, and T scores were obtained at each follow-up. The scores were comparable up to 12 weeks. But by 24 weeks, the T score of group B had improved to -2.86±0.26 in comparison to group A with a score of -3.29±0.53 (p=0.074), suggesting improved bone density and mass with prolonged treatment with teriparatide therapy (Table 4). As PTH has a narrow therapeutic index, serum levels were also periodically studied in the entire follow-up. There was considerable improvement in serum levels of PTH with prolonged therapy with level changes from 27.74±1.03 pg/dl by 6 weeks to 39.7±1.32 pg/dl without any significant abrupt peaks in serum levels or side effects mandating discontinuation of therapy. Levels in control group A remained almost unchanged during the entire course (Table 4).

**Table 4:** T score and serum PTH level PTH, parathyroid hormone

	Group A (mean±SD)	Group B (mean±SD)	p-Value
T score			
6 weeks	-3.4±0.12	-3.36±0.02	0.45
12 weeks	-3.31±0.46	-3.02±0.04	0.074
24 weeks	-3.29±0.53	-2.86±0.26	0.026
Serum PTH levels (pg/dl)			
6 weeks	22.4±0.86	27.74±1.03	0.66
12 weeks	23.44±1.07	32.2±0.74	0.04
24 weeks	24.02±1.22	39.7±1.32	0.036

No significant malunion, non-union, or any implant-related complication was seen in any patients at the end of 24 weeks. There was one case of aseptic superficial wound infection along the surgical suture line, which subsequently healed with regular dressing, antibiotics, and nutritional supplements. Three cases of grade 2 bed sores were seen (one in group A and two in group B) early in the course of follow-up, which subsequently healed uneventfully. One patient in the teriparatide group developed quadriceps wasting due to inadequate motivation, which subsequently improved with regular counseling and exercises.

## Discussion

Worldwide, hip fractures cause considerable public health burden and challenge to the healthcare system, including poor quality of life, economic constraints, functional loss, and higher mortality rate. Intertrochanteric fracture are fairly common hip fractures seen in the elderly population with osteoporosis and are considered the second most common fragility fractures after dorsolumbar spine fractures in active osteoporotic individuals. Surgical fixation of such fractures has been a long established treatment protocol. Even after a properly executed surgical fixation, the outcomes depend on multiple variables including bone quality, fracture geometry, reducibility, and its stability [6]. Surgical outcomes more often than not are compromised due to poor bone quality [6]. In a race between fracture union, early mobilization, and implant failure, in such osteoporotic elderly patients, there has been a controversial role of pharmacotherapy using osteoanabolic agents such as teriparatide in augmenting fracture union. Promising results in animal trials [16] have prompted human studies where some have also demonstrated a quicker healing rates and better overall outcomes in osteoporotic pelvic and distal end radius fractures with supplemental teriparatide therapy [22].

While two meta-analysis studies have shown enhanced early fracture union rates, radiological healing, and early mobilization [18,23], other studies seem to equally disagree. Huang et al. [24] reported better functional outcome at three and six months postoperatively in their study after giving teriparatide in proximal femur fractures, while Rana et al. [14] did not show any results signifying better functional scores at similar follow-up. Kim et al. [25-26] conducted two separate studies with conflicting results evidenced by improvement in the Harris hip score and visual analog scale scores as early as two months in one study with a larger sample size, but no such improvement was seen in the other group with a smaller sample size. Such a controversy highlighted the need for a well-designed study to further substantiate the evidence. While previously, Harris hip score, lower extremity functional score, and short form-12 physical component scale were used in various studies, we decided to proceed with Palmer and Parker mobility scores to assess the functional outcomes, and RUSH score with tip-apex distance calculation, varus collapse, and femoral neck length measurement was used to assess radiological outcomes.

Chesser et al. [27] conducted a short-term study in a large study group, where they demonstrated the efficacy of teriparatide in intertrochanteric fracture union. They achieved full union in all patients by 12 weeks in the study group. In our study also, the members of the study group achieved fracture union earlier with nearly 56.25% of members achieving union as early as 12 weeks and 100% union in all members by 24 weeks and earlier.

The desired outcome after an osteoporotic intertrochanteric fracture treatment is directly related to the capability of the used implant to maintain the fracture reduction until it achieves union. The poor quality of bone often compromises the desired outcomes in the form of fracture collapse in varus angulation, helical blade migration, or femoral neck collapse and shortening. Similar to the study by Huang et al. [24], in our study, we also found significant difference in migration of tip of the blade and femoral neck shortening. Although the difference was less in early follow-up, it turned out to be statistically significant by 24 weeks, suggesting the importance of teriparatide in achieving earlier and stronger bone union and preventing implant-related complications such as screw/blade cutout and implant breakage. This fact was further strengthened by findings of significantly lesser varus collapse by 24 weeks seen in the study group of about 2.08 degrees in comparison to the control group.

Malouf-Sierra et al. [28] found that regular daily subcutaneous injections of 20 micrograms of teriparatide improve overall bone density at the lumbar spine by six months compared to the hip, where visible improvements took almost 12 months of therapy. But surprisingly, our patients performed better, and significant improvements in T score were noted in the study group by 24 weeks of daily therapy, proving the fact that regular teriparatide therapy with additional calcium and nutritional supplements improves bone mass and density, helping in better functional returns.

The higher RUSH and Palmer-Parker scores obtained in our present study point toward improved painless mobilization of patients and improved overall activity levels in the study group. This can be ascribed to quicker fracture union rates and improved bone density and strength in patients receiving the therapy, enabling them to start early weight bearing on the fractured limb, thereby enhancing and improving the general functional status of the patient. Shortcomings of the studies reporting no significant radiological signs of fracture healing could be due to small sample size, short term (six/eight weeks of therapy), and the weekly subcutaneous therapy regime, while in our study, enhanced score may be due to the long term (24 weeks’ therapy) and daily subcutaneous regime as supported by other studies.

In spite of wholehearted efforts and meticulous follow-up, there are multiple limitations associated with our study that must be conceded. First is the limited size of study sample size and the relatively short follow-up, which makes it difficult to conclude regarding secondary outcomes at a later date or when involving larger study group including follow-up issues. Secondly, due to ethical considerations, our study was not properly blinded. Thirdly, no consideration or comparison of the effects of combination therapies with bisphosphonates or newer drugs such as denosumab for osteoporosis management was undertaken. Lastly, the assessments were done at monthly intervals, and therefore the exact time of union could not be assessed precisely, which might be earlier than our follow-up dates.

## Conclusions

We conclude that teriparatide accelerates fracture healing and improves bone mass density in osteoporotic Intertrochanteric hip fractures. Although teriparatide therapy significantly increases the cost of treatment, but the additional benefits in the form of earlier union rates and greater improvement in overall bone density and mass and functional outcome tip the balance in its favor. With an additional advantage of significant improvement in overall bone health and not just at fracture site and the possibility of reduction in the incidence of new and subsequent fragility fractures, our study concludes by recommending the use of teriparatide in osteoporotic intertrochanteric femur fractures. However, we also acknowledge the limitations of our study that could be improved with greater sample size and longer duration studies including focus on other types of fractures.
